# Central Dental Association of Northern New Jersey

**Published:** 1885-02

**Authors:** 


### CENTRAL DENTAL ASSOCIATION OF NORTHERN NEW JERSEY.

### REGULAR MONTHLY MEETING HELD AT THE RESIDENCE OF DR. F. C. BARLOW, JERSEY CITY.

REPORTED FOR THE INDEPENDENT PRACTITIONER.

President Barlow in the chair.

After the usual routine business had been accomplished, Dr. E. Parmly Brown read a paper, entitled "The Standard of Work on the Natural Teeth that the Dentist of the Future Must be Equal to." (See page 66.)

*Dr. W. H. Atkinson*—The ideal future filling is over twenty-five years old in pronouncement. It is a filling of any non-conducting substance, that shall restore the natural contour of the tooth so nearly as to meet the requirements of the artistic type presented in the paper. Then the edges are to be treated precisely as the paper directs, by cutting away just enough of the tooth substance to admit sufficient hold to afford the required strength to the tooth; the gold is then to be built around so as to have a complete shell that shall represent the contour of the tooth, plus enough to admit of finishing, which requires the use of a wedge for separating the teeth a little more than they will be apart when finished; just enough more to admit of the perfect finishing of the knuckle. One thing that I admire very much is knuckling. All of you who are anatomists will see that that is simply following nature. That is the ideal filling. To fully explain how to do that would lead me into too much discursive discussion. You may do it in any way that will accomplish the result that is indicated in the paper. Remember this, that the welding of gold is the annihilating of the spaces between its particles, and it may be done either by the former hand-pressure, or the various kinds of mallets; it does not make a bit of difference where it comes from, if only you get the right impact to make the gold solid.

*Dr. C. S. Stockton*—I do not often like to differ with my friend Dr. Atkinson, and I do not know that I do so now. I believe, as he does, that we should not expect or require a pony to carry more than it can. But there is a great deal of truth in what Dr. Brown has said in regard to the implements to be used, notwithstanding what Dr. Atkinson said with respect to instruments and their impact. There are teeth on which you cannot use a heavy lead mallet, that you can fill and save with the electric mallet, or with what I consider a better instrument, the Bonwill mallet, and that is always ready. It makes as good a blow as the electric-mallet, and I think a better one. You can work around thin walls with that instrument, and make a perfect filling that will support and preserve the tooth; and you can hardly do that with the four hundred pounds' weight that you put on your pony.

*Dr. Atkinson*—Four hundred pounds or an ounce makes no difference; you can strike just as easy and light a blow with one as with the other. It is all flummery to talk about the impact of a hammer being necessarily heavy. I have seen a machinist have such perfect control of a trip-hammer that weighed a ton, that he would just crack the shell of an egg with it, not breaking it.

*Dr. Stockton*—That is true, because the machinery does it, and you can control it; but in using a heavy hand mallet your assistant does not know how frail the walls of the tooth are, nor just how to adapt the weight of the blow to the necessities of the case, and it is not all flummery to say that the blow is likely to be too heavy. I claim that with an instrument that strikes a light blow one can build better than with an instrument that strikes a heavy one. If you have a strong foundation it does not make so much difference what mallet you use, or how heavy a blow you strike; but there are teeth that come into your hands upon which you cannot use any such instrument with safety to the tooth or comfort to the patient. So it is necessary that a little discrimination be behind the trip-hammer. With the electric mallet trip-hammer, or the Bonwill, you can check the impact and take the hammer away. But you should use that which your judgment tells you is the best.

I wish I knew something about this Herbst method, aside from reading. I have read thoroughly what Dr. Bödecker has written respecting it, and from that theoretical knowledge I agree with Dr. Brown, in that I do not think it is practicable. There is too much flummery about it. I think the making of a matrix, where the approximal teeth are out, would be difficult. I do not believe that method will ever be practiced to any extent in this country. I have examined some of the fillings that have been made by Dr. Bödecker, and they look well, but I do not believe the Herbst method will ever become the settled practice of this country. A great deal depends upon the gold used. I understand the German gold is very soft, and works better than our own.

There is a great deal in time saving. If I applied the rubber dam as frequently as some dentists do, the receipts of my practice would be cut off one-third. I fill a great many teeth without applying the rubber dam; I can almost fill the tooth in the time that some dentists use in applying the rubber dam to it. It is one of the most valuable appliances we have in dentistry, and I bless the man who invented and brought it to our notice, but it is used too exclusively by many practitioners. By avoiding its use you not only gain time, but promote the comfort of the patient. A great many patients get tired of the rubber dam; they do not like to be gagged and have their mouths kept open and their tongues useless for hours at a time. Therefore I do not use it when I can do without it. I know I have been successful in a great many cases without its use, yet no one has a higher appreciation of that ingenious appliance than myself.

*Dr. Atkinson*—The Herbst method is not fairly before us yet, and we are going off at half-cock in discussing its merits until we shall have seen more of it than we have seen. I have been asked about it a great many times, and I have said I had no opinion to express. It is but a baby yet. I can speculate, probably, as well as most men, but I do not think it is fair for us to speak of a thing of which we know almost nothing, with the same degree of decisive assertion that we do of those things that we do know about.

*Dr. S. C. G. Watkins*—I am sorry that there is not some person here who is well posted in regard to the Herbst system, for I would like to see the other side of the case presented. Such a defense of the method might bring out points that will not now be broached. I know very little about it myself. I had a tooth filled in New York last winter, the filling being begun with the Bonwill mallet, which was changed for a small hand mallet; and I think the pain and discomfort caused by the blows of that little hand mallet were about the worst I ever suffered in my life. The hand mallet was changed for the automatic mallet, and the filling was finally completed with what might, I suppose, be called the Herbst method; a small blood-stone, revolving in a dental engine, being used to pack the gold. The gold appeared to cohere perfectly, and made a solid and beautiful filling. But that lead mallet! As I think of it now that tooth pains me! The burnishing part of the operation, or the Herbst method, was not at all disagreeable. It had a nice, pleasant feeling, and the next tooth I have filled will be filled by the Herbst method. I think it brings less strain upon the tooth than any other method of packing gold. With regard to solidity or density, I do not think it is necessary to have a filling as thoroughly condensed all through, from beginning to end, as Dr. Brown has stated in his paper; and I think the Herbst method would be admirably adapted to the filling of teeth that would not bear the force necessary to be applied by the old methods.

With regard to applying the matrix, and the difficulty spoken of by Dr. Stockton, I do not think it is necessary to restrict one's self to any particular style of matrix. A matrix can be quickly made of a strip of thin copper tied to the tooth with floss silk; and it does not make any difference whether the tooth stands alone or in an entire arch. Such a matrix can be applied in a few minutes. No great degree of skill is required to do it, and I think it would have a great advantage over the steel matrix described by Dr. Bödecker.

Respecting Dr. Stockton's statement that a great deal of time is wasted in applying the rubber dam, I beg to differ with him. I use the rubber dam more and more every day, and I think it saves time instead of wasting it. I can usually put a rubber dam on a molar tooth as quickly as I can fold and put in a napkin, and I know my filling will be better if I use the rubber dam than it is possible for it to be without it. I believe if dentists would use the rubber dam more, and get more accustomed to its use, they would almost entirely abandon napkins.

*Dr. E. Parmly Brown*—The making of a matrix for the purpose of putting a gold filling into a natural tooth will, I think, never be the general practice. In looking back over the history of dentistry, I know of no operator who has attained excellence and made a reputation who practiced the using of matrices in building up teeth with gold.

*Dr. Atkinson*—Dr. Louis Jack has been doing it for twenty years or more.

*Dr. Brown*—If he has been doing that in this country for a quarter of a century, and I did not find it out, it is very funny; and if he invented such a valuable matrix twenty years ago, and the profession are not using it to-day, it is very funny that so good a thing has been so long in being found out.

*Dr. B. F. Luckey*—I believe there are a great many ways of doing some things. If we want density, and have the time to get it, the electric mallet is, *par excellence,* the thing to use. It is a great favorite of mine; but that we should use it in all operations, whether approximal or large crown cavities, I do not believe is good or profitable practice; it takes too much time. We all know that equal density throughout all parts of the filling is not necessary in all fillings. There are thousands of fillings that were put in twenty or thirty years ago that are not of equal density throughout, and yet they saved the teeth. We can do the same thing in dense teeth to-day. If we are going to build up and contour a large filling in a weak tooth, we must take time and use instruments that will give us the greatest density in the filling. In that I agree with Dr. Brown. That the electric mallet is the most pleasant to the patient I have not found. I have used it for a number of years, in conjunction with various other mallets, and I have found that it is most disagreeable; the patients object to it more than they do to the hand mallet, or the Bonwill mallet, or the automatic mallet. I simply desire to place myself on record as opposed to the idea that we should use but one method. I like to see thorough work, and I like Dr. Brown's idea of what thorough work is, but I do not admit that there is only one way of doing it.

*Dr. Brown*—Dr. Luckey errs in assuming that I advocate doing things in one way only. I claim that there are certain features in the electric mallet which, in my experience, are advantageous. He says the electric mallet is not pleasant. I must not judge by his experience, but by my own. I have witnessed Dr. Webb's use of the electric mallet, and many patients made a great objection to the pain or unpleasant feeling produced by it. But Dr. Webb used it much stronger than was necessary, and with too rigid a plugger. The pluggers I use now are elastic spring pluggers. In nearly four years' use of the electric mallet I can only think of one patient who made the slightest objection to the force of its blow. I am using it every day, almost continuously from morning to night, six days in the week, upon the teeth of little children, middle-age and old people, and delicate, sensitive ladies, and they do not make any objection to it.. A dentist who would continue to use an instrument that his patients made serious objections to, would do a foolish thing.

*Dr. G. S. Meigs*—Some years ago I put in a very large filling in an inferior molar for Dr. William C. McFarlan, and it was reported by the clinic committee that the patient went to sleep while the operation was being performed at the clinic. I recently filled a tooth for a Wall-street gentleman, who made a great deal of fuss about the pain. Afterwards he insisted on my building down a large cuspid, and I told him I would not do it unless I used the mallet. I went to work, and, to my surprise, he slept all through the operation.

*Dr. Brown*—I have a patient, a lady, who has a cleft palate and hare lip. The osseous structure of the superior maxilla is divided at about the median line, into two distinct parts. Some attempts have been made to remedy the trouble, and an artificial palate has been inserted. She has abandoned all these contrivances, and I propose to make a sort of compromise. The left upper central incisor is absent; it was omitted, as well as the bone of the maxilla, in the development, and the other central, which is forward of the lateral, is dead and very black, giving her a bad expression. To regulate these teeth would be a very difficult matter, and if it were done the black central would be unsightly. I wish to know whether it would be safe to extract the two teeth that border on the place of the lost bone. I spoke to a physician about it, and his opinion was that there would be no danger. The appearance of the lady would be very much improved by the removal of the two teeth that are exposed by the hare lip. The missing tooth has been replaced by one on an artificial plate, and if I can with safety take out the two others, the lady's appearance would be very much improved, by substituting three artificial teeth upon the plate. The bone of the superior maxilla is entirely separated, and you can move either side without moving the other.

*Dr. Atkinson*—Remove the teeth by holding firmly the side of the maxilla they are in, giving the forceps a wringing or rotary motion.

*Dr. C. A. Timme*—I have recently used the Herbst method of packing gold. I tried it a year ago, and at that time it did not work satisfactorily, but since Dr. Bodecker returned from Europe, last summer, I have used some of Wolrab's German gold, which is very soft and pure, and I find it works much better. I think the success of the method is due to the quality of the gold, more than to anything else. We have never had pure gold here, in my opinion. I think an agate-point instrument would work better than steel. I polish that kind of fillings with an agate stone. This method has not been used long enough to determine how the fillings will wear.

*Dr. Luckey*—Within the last month or two there has been a great deal of commotion in the medical world over the discovery of a new anæsthetic, the hydrochlorate of cocaine. It has been used in several ophthalmic operations, and it is claimed to be a perfect local anæsthetic in all operations upon mucous surfaces, especially of the eye. I was fortunate enough to obtain half an ounce of the two per cent. solution, thinking it might prove to dentists what they have for years been looking for, something that would, by acting as a local anæsthetic, enable us to prepare cavities in sensitive teeth without pain. I used this cocaine in every way I could think of, dry and wet, with and without a rubber dam, hot and cold; and so far, with this weak solution, I have had no success whatever. Dr. Atkinson tells me to-night that he has had success with a four per cent. solution on very sensitive teeth. I would like to excite the interest or curiosity of the members of this society enough to induce them to investigate the matter, each one for himself, to find if there is any method by which this preparation can be used so as to bring about the results in every case that we would like to have, and that it is claimed this agent will produce.

The ophthalmic surgeons are very jubilant over the success of this anæsthetic in their specialty. They have performed a number of operations upon the eye with perfect ease and without the slightest pain, and without making the patient unconscious. It is also claimed that other painless operations have been performed by its aid. There was an operation performed with it last week in the mouth, either upon the tongue or the inside of the cheek, and there have been operations in the vagina, all without pain. If these things are true, it seems to me that there is something in it for us. It would be a grand thing for us if we could, by the use of such a simple and harmless local anæsthetic as this is said to be, be enabled to prepare cavities painlessly; and, therefore, I bring the subject up, in the hope that each one of you may investigate and experiment with it, and we shall then soon discover what value there is for us in this preparation.

*Dr. Watkins*—Dr. Carr reported, before the First District Society in New York last week, that he had tried this anæsthetic in a great many cases, and in every case it was a perfect success. He used the four per cent. solution. He had been able to touch a nerve, I believe, without causing any pain, and he believed that after a while we will be able to extract nerves painlessly by the use of this anæsthetic. At the same meeting Dr. Woodward stated that he had used the two per cent. solution in a number of cases with no effect.

A motion to pass the subject was carried, and the meeting adjourned.

